# One‐stage Debridement *via* Oblique Lateral Interbody Fusion Corridor Combined with Posterior Pedicle Screw Fixation in Treating Spontaneous Lumbar Infectious Spondylodiscitis: A Case Series

**DOI:** 10.1111/os.12562

**Published:** 2019-11-07

**Authors:** Yong‐jun Tong, Jun‐hui Liu, Shun‐wu Fan, Feng‐dong Zhao

**Affiliations:** ^1^ Department of Orthopaedics, Sir Run Run Shaw Hospital School of Medicine, Zhejiang University Hangzhou China; ^2^ Key laboratory of Musculoskeletal System Degeneration and Degeneration Translational Research of Zhejiang Province Hangzhou China; ^3^ Department of Orthopaedics, Zhejiang Hospital, Hangzhou Zhejiang China

**Keywords:** Anterior lumbar interbody fusion, Extremal lateral lumbar interbody fusion, Lumbar pyogenic spondylodiscitis, Oblique lateral interbody fusion corridor, Vascular and nerve injury

## Abstract

**Objective:**

Surgery is indicated when antibiotic treatment fails in pyogenic spondylodiscitis, which is caused by pathogens such as the *Staphylococcus* species. The aim of the present study was to investigate the efficacy and safety of the oblique lateral interbody fusion (OLIF) corridor approach combined with posterior pedicle screw fixation for treating pyogenic spondylodiscitis.

**Methods:**

This was a retrospective case series study. A total of 11 patients with an average age of 60.7 years (range, 40–70 years; 10 males and 1 females) with lumbar pyogenic spondylodiscitis who underwent single‐stage debridement and reconstruction using the OLIF corridor combined with posterior pedicle screw fixation were recruited in our study from June 2016 to July 2017. All patients had single‐level pyogenic spondylodiscitis between T_12_ and L_5_. The baseline data, perioperative outcomes (operative time, intra‐operative blood loss, and intra‐operative complication), postoperative laboratory tests (erythrocyte sedimentation rate [ESR], C‐reactive protein [CRP], white blood count [WBC], and tissue culture results), long‐term complications (recurrence, fixation failure, and bony non‐fusion rates), and duration of antibiotic administration were reviewed. Outcomes evaluated using a variety of scales including visual analog scale (VAS) score and Oswestry disability index (ODI), were compared pre‐operatively and post‐operatively.

**Results:**

The mean follow‐up period of time was 18.3 months. The average operative time and intra‐operative blood loss were 217.0 ± 91.91 min and 220.9 ± 166.10 mL, respectively. There were no intra‐operative complications, except in 1 patient who encountered somatosensory evoked potentials changes and 1 patient who had motor evoked potentials changes, both without post‐surgery neurological deficits. Causative organisms were identified in 4 patients: *Staphylococcus aureus* in 1 patient and *Streptococcus* in 3 patients. At approximately 8.8 weeks after surgery, WBC, CRP, and ESR had returned to normal levels. All patients were pain free with no recurring infection. There was no fixation failure during follow up. Solid bony fusions were observed in all cases within 6 months. At the final follow up, the mean VAS (0.6 ± 0.69) and ODI (14.4 ± 4.27) were significantly lower than those before surgery (*P* < 0.05).

**Conclusion:**

One‐stage debridement with autogenous iliac bone graft through the OLIF corridor combined with posterior pedicle screw fixation is effective and safe for single‐level spontaneous lumbar pyogenic spondylodiscitis after antibiotic treatment fails.

## Introduction

Spondylodiscitis is an infection that is associated with the destruction of the intervertebral disc. Pyogenic spondylodiscitis is caused by pathogens such as the *Staphylococcus* species, *Escherichia coli*, and *Mycobacterium tuberculosis*. It is a rare condition, accounting for only 2%–7% of all osteomyelitis^1^. However, lately, with the increase in the number of spinal surgeries, the incidence of this condition is also on the rise 2.

Typically, pyogenic spondylodiscitis is quite difficult to treat because the positive rate of the bacterial culture is low. Nam *et al*.^3^ and Sundararaj *et al*.^4^ report a 50%–60% positive rate in cultures obtained by open or fine‐needle aspiration, while Valancius *et al*.^5^ report a much lower rate of approximately 10% from fine needle biopsy. Without a confirmed positive culture, only empiric treatment with antibiotics can be used, which might have inappropriate results. Second, such infections often produce epidural abscesses or neurological symptoms, which cannot be addressed by antibiotics alone 6. In a systematic review, Rutges *et al*.^7^ report that treatment with antibiotics alone is ineffective in approximately 10% of patients, making surgery necessary.

Tsai *et al*.^8^ report that early active debridement followed by treatment with antibiotics achieves better therapeutic results in cases of pyogenic spondylodiscitis. Surgical treatment is reserved for cases in which conservative treatment has failed or neurological deterioration is rapid^9^. However, the best surgical approach remains controversial. An anterior only approach debridement, a posterior only approach debridement, and a combined anterior–posterior debridement have been reported^1^, ^10^, ^11^, ^12^; however, these approaches are associated with certain risks. Debridement through the posterior only approach damages the posterior structure, which finally results in instability of the spine and greater loss of sagittal balance 13. The anterior approach has better clinical outcomes through preserving the posterior structure^14^. Conventional anterior lumbar interbody fusion (ALIF) has the potential for visceral and vascular injury, while the extremal lateral lumbar interbody fusion (XLIF) approach is associated with lumbar plexus injury risk^15^.

Oblique lateral interbody fusion (OLIF) surgery was first performed by Mayer in 1997^16^. The OLIF approach accesses the spine between the abdominal anterior vessel and the psoas muscle (PM). Lumbar plexus and vessel injury are unlikely as dissection is performed between the vessel and the psoas muscle^15^. Thus, the OLIF approach is considered the solution to the limitations of ALIF and LLIF/XLIF^17^. The OLIF surgical approach has been widely used in lumbar disease^18^ but has never been reported as a treatment approach for lumbar pyogenic spondylodiscitis.

In the present study, we retrospectively reviewed cases of single‐level spontaneous pyogenic lumbar discitis that were treated in our hospital from July 2016 to June 2017 using the OLIF corridor combined with posterior internal fixation. The aim of this study is to: (i) introduce a new approach (OLIF combined with the posterior approach) for treating pyogenic spondylodiscitis; (ii) investigate the efficiency and safety of the OLIF corridor approach in treating pyogenic spondylodiscitis; and (iii) analyze the surgical sequence safety of one‐stage debridement an OLIF corridor combined with posterior approach in treating pyogenic spondylodiscitis. We found that one‐stage debridement with autogenous iliac bone graft through the OLIF corridor combined with posterior pedicle screw fixation is effective and safe for single‐level lumbar pyogenic spondylodiscitis.

## Materials and Methods

### *Inclusion and Exclusion Criteria*

Inclusion criteria were as follows: (i) diagnosis of single‐segment pyogenic spondylodiscitis within the T_12_ to L_5_ areas; (ii) pyogenic infection but not tuberculosis (TB); (iii) spontaneous pyogenic spondylodiscitis with no apparent cause; (iv) progression, persistence, or recurrence of the disease despite a minimum of 6 weeks of antimicrobial therapy^9^; (v) surgically treated using one‐stage anterior debridement, interbody fusion with autogenous iliac bone graft through the OLIF corridor, and posterior pedicle screw fixation using the Wiltse approach; (vi) with a minimal follow‐up time beyond 12 months; and (vii) retrospective study.

Exclusion criteria were as follows: (i) infection diagnosed as TB; (ii) secondary pyogenic spondylodiscitis; (iii) treatment with other approaches; and (iv) postoperative follow‐up time less than 12 months.

### *Included Patients*

A total of 21 patients diagnosed with pyogenic spondylodiscitis were enrolled in our hospital from July 2016 to June 2017. Of these, 4 patients did not fulfill the inclusion criteria and 6 patients were lost to follow‐up.

Lumbar pyogenic spondylodiscitis was confirmed in these patients on the basis of the following: back pain or leg pain accompanied by fever; elevation of laboratory tests (white blood cell [WBC], erythrocyte sedimentation rate [ESR], and C‐reactive protein [CRP]); and the results of X‐rays, CT scans, and MRI. These results were also confirmed by histopathological examination regardless of the bacteriological culture results^9^.

### *Surgical Procedure*

#### *Pre‐surgery Preparation*

Preparation for surgery comprised X‐rays, CT scans, and MRI of the lumbar spine. A cross‐sectional MRI was used to determine the operative field between vessels (left side: abdominal aorta [AA]; right side: inferior vena cava) and the anterior medial border of the psoas muscle (PM) at the surgery level^19^.

For the clinically stable patients, antibiotic treatment was discontinued 48 h after the most recent dose had been administered before surgery to improve the positive culture rate. For the clinically unstable patients, the antibiotic treatment was continued until surgery according to the Zimmerli algorithm^20^.

#### *Anesthesia and Surgical Position*

All patients were continuously monitored during surgery using neuro‐electrophysiological methods, such as somatosensory evoked potentials (SEP) and motor evoked potentials (MEP). The surgeries that were performed combined the posterior and OLIF corridor approaches. The posterior fixation was performed first, followed by oblique lateral debridement and interbody fusion. After general anesthesia, the patient was placed in the prone position. After the pedicle screws were inserted, the patient was placed in the lateral position with the nonsurgical side down. The table was adjusted to create a convex bending lumbar spine.

#### *Posterior Approach, Exposure, and Fixation*

A 5‐cm posterior midline incision was made in the target spinal segment, the thoracolumbar fascia was opened and entered using the Wiltse approach^21^, and the facet joints and base of the transverse process were exposed using blunt dissection. The entry point was determined according to the Roy‐Camille method^22^. The pedicle screws were inserted into the affected vertebral bodies. If the intention was to resect more than half of the involved vertebral body, fixation was extended to the cephalad or caudal adjacent vertebrae with posterior fusion to ensure adequate fixation strength.

Pedicle screws were inserted and contoured rods were attached. If there was obvious local deformity, it was corrected by installing contoured rods and with appropriate distraction of the intervertebral space at the involved level.

#### *Oblique Lateral Interbody Fusion Corridor Approach and Exposure*

The patient was placed in a lateral position with the nonsurgical side down. The affected intervertebral space, the inferior edge of the rib, and the iliac crest were marked using fluoroscopy.

A 4‐cm oblique incision parallel to the fiber of the external oblique abdominal muscle was made 3–4 cm anterior to the center of the affected segment. The external oblique, the intra‐abdominal oblique, and the transverse abdominis muscles were bluntly dissected in the direction of their fiber orientation, and the retroperitoneal space was exposed.

The surgeon used the fingers to directly separate the peritoneal tissue. The ureter and vascular structures were identified and protected during dissection and the PM was used as an anatomic landmark. Two long, straight hooks were used to retract the abdominal organs, vessel (left: AA; right: inferior vena cava), ureter, and peritoneal tissue anteriorly to expose the PM. The anterolateral attachments of the PM were bluntly dissected from the lateral circumference of the vertebrae and discs using a periosteal elevator while carefully protecting the sympathetic chain and ensuring that the dissection was not extended to the posterior of the pedicle entrance so as to avoid irritation to the lumbar nerve root. Another two long straight hooks were used to retract the free PM posteriorly, thus finally exposing the lateral border of the vertebrae.

In some cases, para‐spinal abscesses infiltrated the PM, causing it to swell and adhere to the structure around the spine. These cases required extra care when exposing the area. An incision was made between the PM and the vertebral body to drain the abscess, after which a periosteal elevator was used to dissect the PM posterior along the surface of the vertebral body.

#### *Resection and Reconstruction*

Then infectious lesions and all infected granulation tissue, devitalized discs, and sequestra were meticulously debrided, followed by precise curettage to the healthy boundary. The extent of debridement was determined by both the pre‐surgery MRI and findings during the procedure. A healthy boundary was defined as the border between the sequestrum and bleeding cancellous bone. The debrided specimens were submitted to the laboratory for gram staining; aerobic, anaerobic and fungal culture; and histopathological examination.

The debridement field was extensively irrigated with hydrogen peroxide, povidone iodine, and normal saline. After irrigating, the surgical instruments were replaced with new ones. The vertebral body defect was then measured, and an autologous bone graft of appropriate length that was harvested from the iliac crest was punched into the defect to reconstruct the anterior column and create the interbody fusion.

#### *Post‐surgery Treatment*

After the surgery, broad‐spectrum intravenous antibiotics were modified for use according to the culture sensitivity test. In culture‐negative cases, the previous antibiotics regime was continued. Antibiotics were used until the patient's clinical symptoms were relieved, and the inflammatory factor had returned to normal^23^.

The patient was able to ambulate immediately with the protection of a waist brace and had to be careful not to twist or bend during the first 3 months after the procedure.

### *Outcome Measurements*

The basic characteristics of recruited patients were recorded, including gender, age, symptoms, and the infected vertebral interval level.

#### *Peri‐operative Outcomes*

Peri‐operative outcomes include operative time (from the beginning of skin incision to surgical closure), intra‐operative blood loss (measured by the bleeding volume at the gauzes), and peri‐operative complications (SEP or MEP changes, vessel injury, nerve injury, and ureter injury).

#### *Peri‐operative Laboratory Tests*

Peri‐operative laboratory tests included erythrocyte sedimentation rate (ESR), C‐reactive protein (CRP), white blood count (WBC), and tissue culture results. WBC, CRP, and ESR levels were assessed weekly after surgery until the results returned to normal. Cultures were also taken for bacteria and fungi.

#### *Clinical Outcome Measurements*

The visual analogue scale (VAS) scoring system was used to evaluate the pre‐surgery and post‐surgery pain level of patients. VAS scores were from 0 = no pain to 10 = very intense pain.

The Oswestry disability index (ODI) is one of the most commonly used condition‐specific outcome measures for spinal disorders^24^. The ODI score system includes 10 sections: pain intensity, personal care, lifting, walking, sitting, standing, sleeping, sex life, social life, and traveling. For each section, the total score is 5. The score is calculated as follows: total score/(5× number of questions answered) × 100%.

Pre‐surgery and post‐surgery pain and disability were evaluated using the VAS score and the ODI.

#### *Imaging Measurements*

All patients underwent X‐rays, CT, and MRI of the lumbar spine before surgery and X rays of the lumbar spine 1 day after surgery and again 1, 3, 6, and 12 months after surgery, and a CT scan was performed 12 months after surgery to estimate long‐term complications, including recurrence, fixation failure, and bony non‐fusion rates.

The extent of bone graft healing was evaluated using the method introduced by Santos *et al*.^25^, which indicated the presence of bony trabeculation or lack of bone lucency at the bone graft/vertebral body interface.

The absence of infection was defined as having no fever, pain, or graft bone union at the interface 12 months after surgery^26^.

#### *Statistical Analyses*

Statistical analyses were performed using SPSS 16.0 software (SPSS, Chicago, IL, USA). The paired *t*‐test was used to assess the difference between the pre‐surgery and post‐surgery VAS scores and ODI. A *P*‐value < 0.05 was considered statistically significant.

### *Results*

#### *Demographic Data*

A total of 11 cases (10 males and 1 female) met the inclusion criteria; the average age was 60.7 years (range, 40–70 years), with a minimum follow‐up duration of 12 months (mean duration, 18.3 months; range 13–24 months). All 11 patients were diagnosed with spontaneous pyogenic spondylodiscitis with no apparent cause; 7 patients complained of severe back pain and 4 others complained of radicular pain. A summary of the clinical characteristics and surgical parameters of the 11 patients is provide in Table 1.

**Table 1:** Clinical characteristics and surgical parameters of 11 patients

Case nuber	1	2	3	4	5	6	7	8	9	10	11	Average
Gender	Male	Male	Male	Female	Male	Male	Male	Male	Male	Male	Male	—
Age (years)	69	69	63	62	40	70	50	52	61	69	63	60.7 ± 9.61
Symptom	Back pain	Back pain	Radicular pain	Back pain	Back pain	Radicular pain	Radicular pain	Back pain	Back pain	Radicular pain	Back pain	—
Level	L_2/__3_	L_2/__3_	L_3/__4_	L_2/__3_	T_12_/L_1_	T_12_/L_1_	L_4/__5_	L_3/4_	L_4/5_	L_4/5_	L_4/5_	—
Mircrobiology	Streptococcus	Streptococcus	Streptococcus	—	*Staphylococcus aureus*	—	—	—	—	—	—	—
Surgery(min)	305	200	180	240	357	80	280	115	145	155	330	217.0 ± 91.91
Blood Loss(mL)	500	150	30	150	300	200	500	200	20	80	300	220.9 ± 166.1
Neuroelectrophysi‐ological monitoring abnormility	—	—	MEP change	—	—	—	SSEP change	—	—	—	—	—
Complication	NA	NA	NA	NA	NA	NA	NA	NA	NA	NA	NA	—
Follow‐up (months)	20	15	24	19	14	24	14	18	13	20	20	18.3 ± 3.88
Duration Of inflammation biomarker returned to normal (weeks)	10	10	11	12	6	8	10	9	6	7	8	8.8 ± 1.99

NA, not applicable; MEP, motor evoked potentials; SEP, somatosensory evoked potential.

#### *Peri‐Operative Outcome*

The average duration and blood loss during surgery were 217.0 ± 91.91 min and 220.9 ± 166.10 mL, respectively.

#### *Peri‐operative Laboratory Tests*

Intra‐operative pus cultures were obtained for all 11 patients. In 3 patients, the causative organisms were identified as *S. aureus*. In 1 patient, the causative organism was *Streptococcus*. The cultures from the remaining 7 patients were negative but evidence of bacterial osteomyelitis was subsequently identified using histopathology.

During an average of 8.82 weeks after surgery, WBC, CPR, and ESR levels in all patients had returned to normal.

#### *Clinical Outcome Measurements*

All patients were relieved of back and radicular pain at finally follow‐up. The mean VAS scores were 6.9 ± 1.4 and 0.6 ± 0.7 before surgery and at 12 months follow‐up, respectively. The mean ODI were 86.6 ± 2.8 and 14.4 ± 4.3 at corresponding time points. Both the VAS and ODI at the final follow‐up examination were significantly lower than those before surgery (*P* < 0.05, respectively) (Table 2).

**Table 2:** Pre and post‐operative visual analog scale (VAS) score and Oswestry disability index (ODI) comparison

Case number		1	2	3	4	5	6	7	8	9	10	11	Average
VAS	Pre‐operative	7	6	8	6	4	8	7	7	8	9	6	6.9 ± 1.4
	Final Follow up	1	0	1	0	0	1	1	0	0	2	0	0.6 ± 0.7*
ODI	Pre‐operative	88	86	90	86	80	88	90	86	86	88	84	86.6 ± 2.8
	Final Follow up	16	16	18	20	8	14	14	12	18	16	6	14.4 ± 4.3*

* *P* < 0.05 compared with preoperative values.

#### *Imaging Measurements*

**Figure 1:**
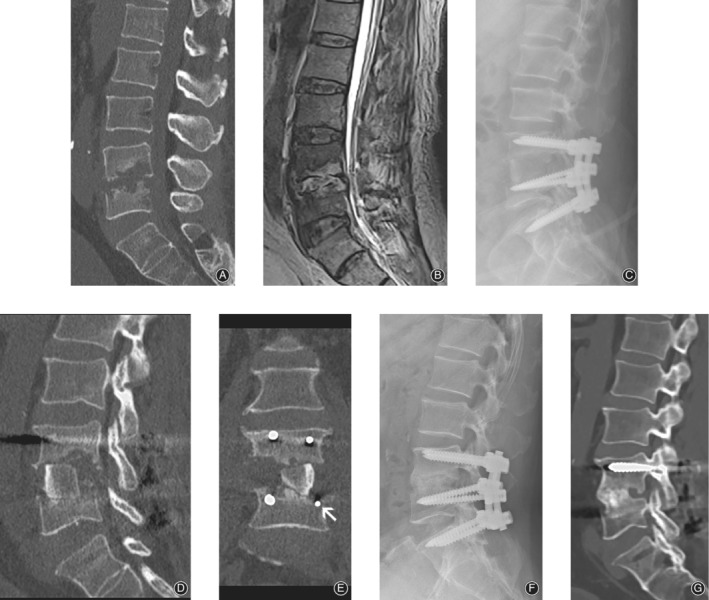
Preoperative CT (A), MRI (B) of a 63‐year‐old man suffering from L_4/__5_ spondylodiscitis with partial destruction of the vertebral bodies. Immediately post‐surgery X‐ray (C) and CT scan (D, E). Both the L_5_ pedicle screws were placed below the cortical vertebrae (E, white arrow). One year after surgery, X‐ray (F) and CT (G) scan showed a solid fusion of the bone graft and vertebral body interface.

**Figure 2:**
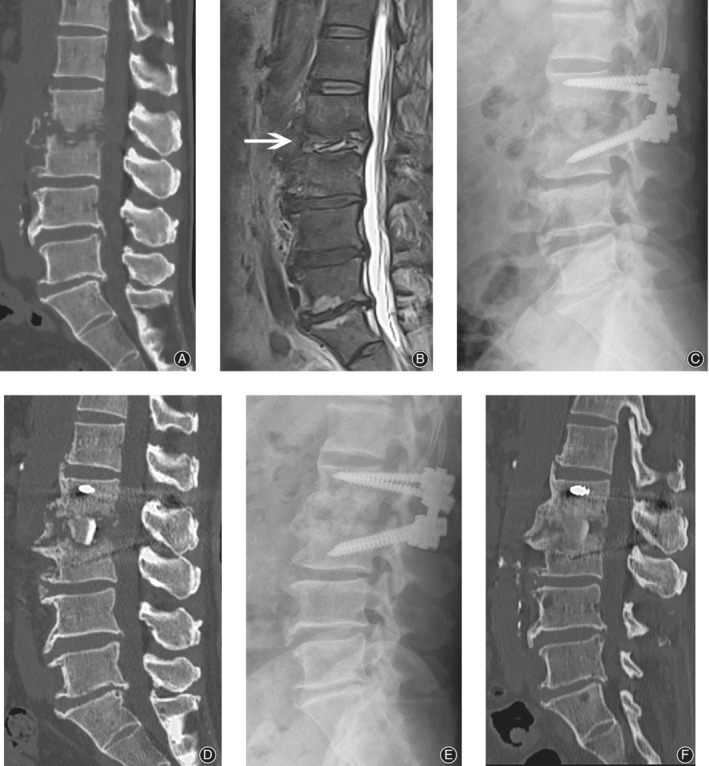
Sixty‐two‐year‐old woman, whose chief complaint was back pain of more than 3 months. Pre‐operative CT (A) and MRI (B) images revealed L_2–__3_ intra‐vertebral space infection with both upper and lower endplate destruction. Debridement and reconstruction underwent *via* oblique lateral interbody fusion (OLIF) corridor and posterior approach. A massive structure bone graft was seen in the immediately postoperative film (C) and CT scan (D). Post‐surgery 1 year, both the film (E) and CT scan (F) showed perfect fusion between bone graft and vertebrae interface. White arrow indicates the index level.

**Figure 3:**
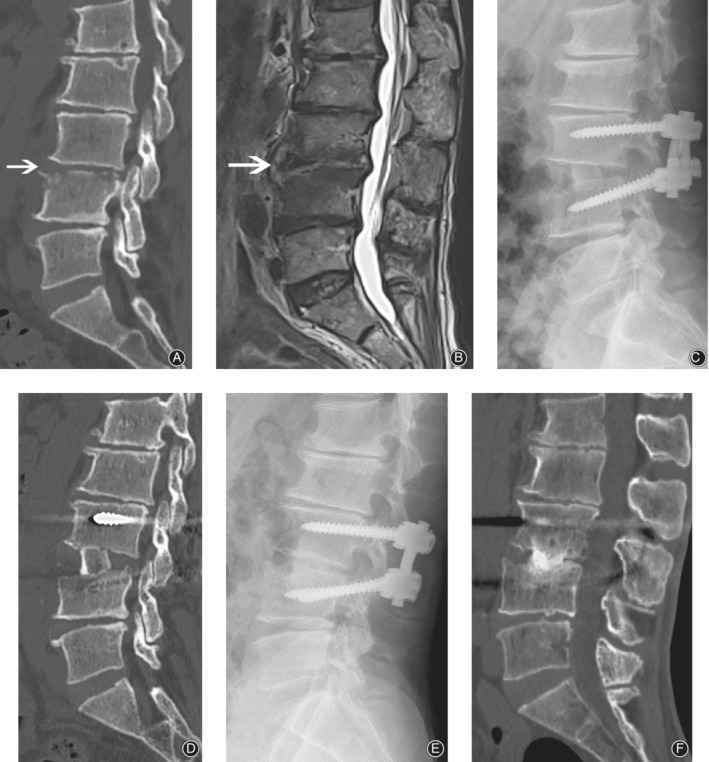
Fifty two‐year old man, with back pain for 2 months without fever. Pre‐operative CT scan (A) showed superior endplate destruction of L_4_. Pre‐operative MRI (B) shows high T2 signal in disc space and low T2 signal in both upper and lower endplate. Surgery was performed *via* as mentioned method. A massive structure bone graft was seen in the immediately postoperative film (C) and CT scan (D). One year after surgery, fusion was achieved between the interface in both film (E) and CT (F) scan. White arrow indicates the index level.

No screw or rod breakage were found in the X‐ray or CT scan during follow up. Solid bony fusions were observed in all 11 patients at the final follow‐up point (Figs 1, 2, 3).

#### *Complication*

##### *Intra‐operative Complications*

Changes in SEP and MEP were observed in 2 patients, both without post‐surgery neurological deficits. No major vessels were injured during surgery. There was no ureter injury or retrograde ejaculation.

##### *Long‐term Complications*

Non‐union was not observed in our cases. All patients were back and radicular pain free at the final follow‐up. In other words, no infections had recurred (as defined by Bernard *et al*.)^26^.

## Discussion

Lumbar pyogenic spondylodiscitis is a rare but serious disease of the spine. The goal of surgical treatment is to debride the infection, relieve the symptoms, reconstruct spinal stability, and obtain microbial specimens^13^. Thorough debridement following bone grafting and internal fixation is the current standard surgical protocol, but the choice of surgical approach, the surgical stage, and the duration of antibiotics administration remain controversial.

Orthopaedists are most familiar with the posterior surgical approach, making it the most common procedure for treating pyogenic spondylodiscitis 27; however, a recent systematic review found that the anterior approach for debridement of the lesion could achieve better clinical results^7^. Many surgeons prefer anterior debridement through the ALIF or XLIF corridor followed by a posterior stabilization procedure^9^; however, these anterior or posterior debriding approaches have certain risks. Finding a safer approach is vital in the treatment of pyogenic spondylodiscitis.

The fairly new oblique lateral approach was first introduced by Mayer^16^ and uses the natural space between the lateral border of the abdominal vessel (left side: aorta (AA) and right side: inferior vena cava), and the anterior medial border of the psoas muscle (PM). This approach has been widely used to treat lumbar disc disease^28^, lumbar spondylolisthesis^29^, degenerative scoliosis^30^, and adjacent segment degeneration^31^; however, its application in lumbar pyogenic spondylodiscitis has not been illuminated.

This study might be the first to report on debridement and fusion through the OLIF corridor combined with placement of posterior pedicle screws in lumbar pyogenic spondylodiscitis for which antibiotic treatment failed with a minimum follow up of 12 months after the procedure.

In our study, there were no severe nerve or vascular injuries during surgery; only MEP and SEP changes were observed during surgeries, which did not have any negative consequences. After 12 months, the follow‐up examinations revealed that VAS and ODI scores had significantly improved over those before surgery. There was no recurrence of infection and 100% consolidated fusion was achieved, which suggests that anterior debridement and fusion through the OLIF corridor combined with posterior pedicle screws is safe and effective in treating lumbar pyogenic spondylodiscitis for which antibiotic treatment has failed.

### *Oblique Lateral Interbody Fusion Corridor Advantages Compared with Posterior‐only Approach*

Anterior debridement and fusion through the OLIF corridor has several advantages over the conventional posterior approach, as outlined below.

#### *Complete Debridement*

The pathoanatomy of pyogenic vertebral osteomyelitis is typically anterior to the neural contents; therefore, the lesion cannot be fully exposed using the posterior approach, which might result in inadequate debridement. Yang *et al*.^32^ and Lu *et al*.^33^ reported 86% and 92% infection control rates, respectively, using the posterior approach alone. Having to use drainage during posterior debriding to reduce the recurrence rate of infection implies that debridement *via* the single posterior approach is not sufficient^34^. To reduce the recurrence rate, many orthopaedists have suggested radical debridement using the posterior approach, which theoretically results in excessive removal of healthy bone^10^, ^35^, ^36^, ^37^. Exposing *via* the OLIF corridor, the lesion can be clearly viewed and debrided up to the healthy bone, which results in precise debridement without any residual lesion or excessive removal of healthy bone. In our study, the infections were eliminated in all patients, which suggested that the lesion was precisely and completely debrided.

#### *Preserving the Posterior Stable Structure*

Some authors have reported performing posterior approach debridement to the anterior lesion in the vertebral osteomyelitis cases^36^, ^37^; however, we question this approach because in cases of infection, the anterior structure of the vertebral body have been destroyed by bacteria, making the spine unstable. To achieve a thorough debridement through the posterior approach, the posterior structures, such as the lamina, the articular process, and the posterior ligamental complex, must be removed, which would further damage the structure of the spine. It has been found that the posterior structures of the spine are essential for preserving the long‐term spinal stability when the anterior column is disrupted^38^. Debridement and fusion *via* the OLIF corridor approach combined with posterior pedicle screw fixation using the Wilste approach completely preserves the posterior spinal structures, which most likely benefits the long‐term spinal stability.

#### *Massive Tricortical Structural Bone Grafting Results in Better Fusion*

Because of the exposure limitations of the posterior surgery approach, it is difficult to select an appropriate graft size to achieve sufficient anterior support. In an OLIF corridor approach, the size of the debridement area can be directly measured, after which the appropriately sized of tricortical autogenous bone graft to be harvested from the iliac crest to fill in the defect can better stabilize the anterior column (Figs 1 and 2), resulting in faster fusion. McAfee *et al*.^39^ found that anterior interbody fusion cages showed an early continuous bone bridge (sentinel sign). Dai *et al*.^40^ and Duarte and Vaccaro^9^ reported that anterior bone grafts fuse faster and more completely. In our study, the bone grafts in all patients were fused within 12 months after surgery, which was comparable to the results presented in previous reports 9, 41.

#### *Decreased Risk of Post‐surgery Central Nervous System Infection*

Some studies have stated that the risk of a dural tear during posterior surgery was from 1.6%^42^ to 8%^43^. Debriding the anterior lesion using the posterior approach invades the spinal canal, which increases the risk of infection to the central nervous system after a durotomy. Because the incidence of spondylodiscitis is low, there are no reports of post‐surgery infections of the central nervous system during spondylodiscitis. Many surgeons prefer not to invade the posterior tissue to expose infected tissue and instead perform an initial anterior debridement followed by a posterior stabilization procedure^9^. We believe that surgery for spondylodiscitis using the posterior approach theoretically increases the risk of intracranial infection when there is a dural tear. Debridement of spondylodiscitis using the OLIF corridor approach results in minimal invasion of the structure of the spinal canal, and the risk of infection of the central nervous system from a dural rupture is theoretically reduced.

#### *Decreased Trauma*

Posterior debridement requires more exposure to spinal tissues and structures; therefore, it is more traumatic and time consuming than anterior debridement. Gorensek *et al*.^35^ reported that the average blood loss during surgery and surgery time were 1150 mL and 207 min, respectively, using the single posterior approach for debridement and fusion of pyogenic osteomyelitis. In our study, blood loss during surgery was 220 mL, much less than that noted in previous studies, while the average surgery duration was 217 min, which was comparable to that noted in previous studies. The results of our study suggest that although the combined anterior–posterior approach added a step to the single posterior approach, it results in less bleeding without an increase in surgery duration.

### *Oblique Lateral Interbody Fusion Corridor Advantages Compared with Anterior Lumbar Interbody Fusion or Extremal Lateral Lumbar interbody fusion*

Debridement and interbody fusion through the OLIF corridor also has advantages over the conventional ALIF or XLIF approach. The conventional approach through the ALIF corridor has less potential for injury to the lumbar plexus; however, it increases the risk of injury to major vessels from 1.9% to 4.6%^44^, ^45^. However, the approach through the XLIF corridor is associated with a lower risk of injury to the blood vessels^46^ but an increased risk of nerve damage, from 3.4% to 42%^47^, ^48^.

Debridement through the OLIF corridor balances these risk factors of the other approaches very well. Different from the XLIF corridor, surgery through the OLIF corridor uses the natural space between the PM and vessels without invading the lumbar plexus in the PM. Silvestre et al. reported that the risk to the peripheral nerve injury was lower in OLIF surgery, accounting for only 2.7%^49^. Lee *et al*.^50^ and Miscuis *et al*.^51^ report that surgery through the OLIF corridor could be safely conducted without electrophysiological monitoring. By monitoring the nerves during surgery in our study, we also confirmed that although two patients had potential nerve changes, there were no neural consequences after surgery. When compared with the conventional ALIF technique, the OLIF corridor approach uses the intervertebral space posterior to the AA without dissecting it, which can, thus, dramatically reduce the risk of injury to large blood vessels^52^. In our study, no significant vascular injury was observed during surgery, which suggests that the OLIF corridor approach is safe for the blood vessels.

### *Surgical Sequence*

Controversy remains over whether internal fixation should be done in a single stage or in two stages after debridement of the infected area. Some surgeons are concerned that one‐stage internal fixation increases the risk of re‐infection^52^, ^53^; however, several clinical follow‐up studies have confirmed that one‐stage debridement combined with posterior internal iliac bone graft^54^ or titanium mesh fusion^55^ did not increase the recurrence rate of infection after surgery.

We completed the surgeries in one stage and preferred posterior pedicle screw fixation followed by anterior debridement and fusion. There were three advantages to this procedure. First, in patients with severe vertebral destruction and kyphosis, the local deformity is corrected through posterior distraction, which also facilitates anterior debridement in narrow space cases. Second, posterior fixation being done first does not directly expose the screws to the infected surgical field, which reduces the risk of infection to the structure. Third, the procedure avoids incorrect screw trajectory. When completing anterior debriding and fusion followed by posterior screw insertion, it is possible for the posterior pedicle screw to be inserted at an incorrect trajectory, making the large bone graft protrude out of the defected vertebral area. This is avoided by performing the posterior fixation first (Fig. 1).

Our study showed that after a minimum of 12 months of follow up, there were no signs of infection and bony fusion was stable, which confirmed that one‐stage anterior surgery debridement and intervertebral fusion through the OLIF corridor and posterior internal fixation is, indeed, useful and does not increase the recurrence rate of infection.

### *Limitations*

There are several limitations of this study. First, the sample number is small: we examined only 11 cases, the results of which might not be generalized. Larger randomized control trials are necessary to analyze the outcomes of the suggested method in treating lumbar pyogenic spondylodiscitis. Second, this is a retrospective study, the nature of which lacks a random assignment of subjects and does not allow for the enrolled patients to undergo different treatment methods for subsequent comparison of clinical outcomes. The benefit of this treatment should be rigorously evaluated using a large patient population with prospectively controlled comparison groups.

### *Conclusion*

Single‐stage debridement with autogenous iliac bone graft through the OLIF corridor and posterior pedicle screw fixation using the Wiltse approach is an alternative method by which to treat pyogenic spondylodiscitis when antibiotics have failed, with less trauma, blood loss, and fewer complications than with the traditional approaches and with comparable surgery duration, which suggests that this approach is effective and safe for treating single‐level lumbar pyogenic spondylodiscitis after antibiotic treatment has failed.

## References

[1] Yaldz C , Ozdemir N , Yaman O , Feran HG , Tansug T , Minoglu M . A retrospective study of 39 patients treated with anterior approach of thoracic and lumbar spondylodiscitis: clinical manifestations, anterior surgical treatment, and outcome. Medicine (Baltimore), 2015, 94: e2110. 10.1097/MD.0000000000002110 PMC5058998 26632729

[2] Kehrer M , Pedersen C , Jensen TG , Lassen AT . Increasing incidence of pyogenic spondylodiscitis: a 14‐year population‐based study. J Infect, 2014, 68: 313–320. 24296494 10.1016/j.jinf.2013.11.011

[3] Nam KH , Song GS , Han IH , Choi BK , Cha SH . Diagnostic value of biopsy techniques in lumbar spondylodiscitis: percutaneous needle biopsy and open biopsy. Korean J Spine, 2011, 8: 267–271. 26064144 10.14245/kjs.2011.8.4.267 PMC4461738

[4] Sundararaj GD , Babu N , Amritanand R , *et al* Treatment of haematogenous pyogenic vertebral osteomyelitis by single‐stage anterior debridement, grafting of the defect and posterior instrumentation. J Bone Joint Surg Br, 2007, 89: 1201–1205. 17905958 10.1302/0301-620X.89B9.18776

[5] Valancius K , Hansen ES , Hoy K , Helmig P , Niedermann B , Bünger C . Failure modes in conservative and surgical management of infectious spondylodiscitis. Eur Spine J, 2013, 22: 1837–1844. 23247861 10.1007/s00586-012-2614-3 PMC3731482

[6] Herren C , Jung N , Pishnamaz M , Breuninger M , Siewe J , Sobottke R . Spondylodiscitis: diagnosis and treatment options. Dtsch Arztebl Int, 2017, 114: 875–882. 29321098 10.3238/arztebl.2017.0875 PMC5769318

[7] Rutges JP , Kempen DH , van Dijk M , Oner FC . Outcome of conservative and surgical treatment of pyogenic spondylodiscitis: a systematic literature review. Eur Spine J, 2016, 25: 983–999. 26585975 10.1007/s00586-015-4318-y

[8] Tsai TT , Yang SC , Niu CC , *et al* Early surgery with antibiotics treatment had better clinical outcomes than antibiotics treatment alone in patients with pyogenic spondylodiscitis: a retrospective cohort study. BMC Musculoskelet Disord, 2017, 18: 175. 28449655 10.1186/s12891-017-1533-1 PMC5408454

[9] Duarte RM , Vaccaro AR . Spinal infection: state of the art and management algorithm. Eur Spine J, 2013, 22: 2787–2799. 23756630 10.1007/s00586-013-2850-1 PMC3843785

[10] Zhang T , Hu J , Wu J , Liu J , Ni S , Duan C . One‐stage posterior debridement and fusion combined with irrigation and drainage for the treatment of postoperative lumbar spondylodiscitis. Acta Orthop Traumatol Turc, 2018, 52: 277–282. 10.1016/j.aott.2018.04.004 PMC6150443 29779968

[11] Lee BH , Park JO , Kim HS , Lee HM , Cho BW , Moon SH . Transpedicular curettage and drainage versus combined anterior and posterior surgery in infectious spondylodiscitis. Indian J Orthop, 2014, 48: 74–80. 24600067 10.4103/0019-5413.125508 PMC3931157

[12] Lin Y , Chen WJ , Zhu WT , *et al* Single‐level lumbar pyogenic spondylodiscitis treated with minimally invasive anterior debridement and fusion combined with posterior fixation via Wiltse approach. J Huazhong Univ Sci Technolog Med Sci, 2013, 33: 707–712. 24142724 10.1007/s11596-013-1184-x

[13] Vcelak J , Chomiak J , Toth L . Surgical treatment of lumbar spondylodiscitis: a comparison of two methods. Int Orthop, 2014, 38: 1425–1434. 24859896 10.1007/s00264-014-2360-8 PMC4071504

[14] Si M , Yang ZP , Li ZF , Yang Q , Li JM . Anterior versus posterior fixation for the treatment of lumbar pyogenic vertebral osteomyelitis. Orthopedics, 2013, 36: 831–836. 23746024 10.3928/01477447-20130523-33

[15] Mobbs RJ , Phan K , Malham G , Seex K , Rao PJ . Lumbar interbody fusion: techniques, indications and comparison of interbody fusion options including PLIF, TLIF, MI‐TLIF, OLIF/ATP, LLIF and ALIF. J Spine Surg, 2015, 1: 2–18. 27683674 10.3978/j.issn.2414-469X.2015.10.05 PMC5039869

[16] Mayer HM . A new microsurgical technique for minimally invasive anterior lumbar interbody fusion. Spine (Phila Pa 1976), 1997, 22: 691–699 discussion 700. 9089943 10.1097/00007632-199703150-00023

[17] Phan K , Maharaj M , Assem Y , Mobbs RJ . Review of early clinical results and complications associated with oblique lumbar interbody fusion (OLIF). J Clin Neurosci, 2016, 31: 23–29. 27349468 10.1016/j.jocn.2016.02.030

[18] Li JX , Phan K , Mobbs R . Oblique lumbar interbody fusion: technical aspects, operative outcomes, and complications. World Neurosurg, 2017, 98: 113–123. 27777161 10.1016/j.wneu.2016.10.074

[19] Molinares DM , Davis TT , Fung DA . Retroperitoneal oblique corridor to the L2‐S1 intervertebral discs: an MRI study. J Neurosurg Spine, 2015, 24: 248–255. 10.3171/2015.3.SPINE13976 26451662

[20] Zimmerli W . Clinical practice. Vertebral osteomyelitis. N Engl J Med, 2010, 362: 1022–1029. 20237348 10.1056/NEJMcp0910753

[21] Wiltse LL , Spencer CW . New uses and refinements of the paraspinal approach to the lumbar spine. Spine (Phila Pa 1976), 1988, 13: 696–706. 3175760

[22] Roy‐Camille R , Saillant G , Mazel C . Plating of thoracic, thoracolumbar, and lumbar injuries with pedicle screw plates. Orthop Clin North Am, 1986, 17: 147–159. 3945476

[23] Lin Y , Li F , Chen W , Zeng H , Chen A , Xiong W . Single‐level lumbar pyogenic spondylodiscitis treated with mini‐open anterior debridement and fusion in combination with posterior percutaneous fixation via a modified anterior lumbar interbody fusion approach. J Neurosurg Spine, 2015, 23: 747–753. 26340382 10.3171/2015.5.SPINE14876

[24] Fairbank JC , Pynsent PB . The Oswestry disability index. Spine (Phila Pa 1976), 2000, 25: 2940–2952 discussion 2952. 11074683 10.1097/00007632-200011150-00017

[25] Santos ER , Goss DG , Morcom RK , Fraser RD . Radiologic assessment of interbody fusion using carbon fiber cages. Spine (Phila Pa 1976), 2003, 28: 997–1001. 12768137 10.1097/01.BRS.0000061988.93175.74

[26] Bernard L , Dinh A , Ghout I , *et al* Antibiotic treatment for 6 weeks versus 12 weeks in patients with pyogenic vertebral osteomyelitis: an open‐label, non‐inferiority, randomised, controlled trial. Lancet, 2015, 385: 875–882. 25468170 10.1016/S0140-6736(14)61233-2

[27] Gala RJ , Bovonratwet P , Webb ML , Varthi AG , Daubs MD , Grauer JN . Different fusion approaches for single‐level lumbar spondylolysis have similar perioperative outcomes. Spine (Phila Pa 1976), 2018, 43: E111–e117. 28591074 10.1097/BRS.0000000000002262

[28] Fujibayashi S , Hynes RA , Otsuki B , Kimura H , Takemoto M , Matsuda S . Effect of indirect neural decompression through oblique lateral interbody fusion for degenerative lumbar disease. Spine (Phila Pa 1976), 2015, 40: E175–E182. 25394317 10.1097/BRS.0000000000000703

[29] Sato J , Ohtori S , Orita S , *et al* Radiographic evaluation of indirect decompression of mini‐open anterior retroperitoneal lumbar interbody fusion: oblique lateral interbody fusion for degenerated lumbar spondylolisthesis. Eur Spine J, 2017, 26: 671–678. 26245906 10.1007/s00586-015-4170-0

[30] Ohtori S , Mannoji C , Orita S , *et al* Mini‐open anterior retroperitoneal lumbar interbody fusion: oblique lateral interbody fusion for degenerated lumbar spinal kyphoscoliosis. Asian Spine J, 2015, 9: 565–572. 26240716 10.4184/asj.2015.9.4.565 PMC4522447

[31] Phan K , Mobbs RJ . Oblique lumbar interbody fusion for revision of non‐union following prior posterior surgery: a case report. Orthop Surg, 2015, 7: 364–367. 26791588 10.1111/os.12204 PMC6583715

[32] Yang SC , Chen WJ , Chen HS , Kao YH , Yu SW , Tu YK . Extended indications of percutaneous endoscopic lavage and drainage for the treatment of lumbar infectious spondylitis. Eur Spine J, 2014, 23: 846–853. 24448892 10.1007/s00586-013-3157-y PMC3960440

[33] Lu ML , Niu CC , Tsai TT , Fu TS , Chen LH , Chen WJ . Transforaminal lumbar interbody debridement and fusion for the treatment of infective spondylodiscitis in the lumbar spine. Eur Spine J, 2015, 24: 555–560. 25228109 10.1007/s00586-014-3585-3

[34] Lee BH , Lee HM , Kim TH , *et al* Transpedicular curettage and drainage of infective lumbar spondylodiscitis: technique and clinical results. Clin Orthop Surg, 2012, 4: 200–208. 22949951 10.4055/cios.2012.4.3.200 PMC3425650

[35] Gorensek M , Kosak R , Travnik L , Vengust R . Posterior instrumentation, anterior column reconstruction with single posterior approach for treatment of pyogenic osteomyelitis of thoracic and lumbar spine. Eur Spine J, 2013, 22: 633–641. 22922802 10.1007/s00586-012-2487-5 PMC3585646

[36] Skovrlj B , Guzman JZ , Caridi J , Cho SK . Posterior‐only circumferential decompression and reconstruction in the surgical Management of Lumbar Vertebral Osteomyelitis. Global Spine J, 2016, 6: e35–e40. 26835214 10.1055/s-0035-1550341 PMC4733378

[37] Endres S , Wilke A . Posterior interbody grafting and instrumentation for spondylodiscitis. J Orthop Surg (Hong Kong), 2012, 20: 1–6. 22535802 10.1177/230949901202000101

[38] James KS , Wenger KH , Schlegel JD , Dunn HK . Biomechanical evaluation of the stability of thoracolumbar burst fractures. Spine (Phila Pa 1976), 1994, 19: 1731–1740. 7973968 10.1097/00007632-199408000-00013

[39] McAfee PC , Boden SD , Brantigan JW , *et al* Symposium: a critical discrepancy‐a criteria of successful arthrodesis following interbody spinal fusions. Spine (Phila Pa 1976), 2001, 26: 320–334. 11224871 10.1097/00007632-200102010-00020

[40] Dai LY , Chen WH , Jiang LS . Anterior instrumentation for the treatment of pyogenic vertebral osteomyelitis of thoracic and lumbar spine. Eur Spine J, 2008, 17: 1027–1034. 18575900 10.1007/s00586-008-0661-6 PMC2518771

[41] Ha KY , Shin JH , Kim KW , Na KH . The fate of anterior autogenous bone graft after anterior radical surgery with or without posterior instrumentation in the treatment of pyogenic lumbar spondylodiscitis. Spine (Phila Pa 1976), 2007, 32: 1856–1864. 17762293 10.1097/BRS.0b013e318108b804

[42] Williams BJ , Sansur CA , Smith JS , *et al* Incidence of unintended durotomy in spine surgery based on 108,478 cases. Neurosurgery, 2011, 68: 117–123 discussion 123–114. 21150757 10.1227/NEU.0b013e3181fcf14e

[43] Ishikura H , Ogihara S , Oka H , *et al* Risk factors for incidental durotomy during posterior open spine surgery for degenerative diseases in adults: a multicenter observational study. PLoS One, 2017, 12: e0188038. 10.1371/journal.pone.0188038 PMC5708748 29190646

[44] Brau SA , Delamarter RB , Schiffman ML , Williams LA , Watkins RG . Vascular injury during anterior lumbar surgery. Spine J, 2004, 4: 409–412. 15246301 10.1016/j.spinee.2003.12.003

[45] Quraishi NA , Konig M , Booker SJ , *et al* Access related complications in anterior lumbar surgery performed by spinal surgeons. Eur Spine J, 2013, 22: S16–S20. 23250515 10.1007/s00586-012-2616-1 PMC3578511

[46] Kueper J , Fantini GA , Walker BR , Aichmair A , Hughes AP . Incidence of vascular complications during lateral lumbar interbody fusion: an examination of the mini‐open access technique. Eur Spine J, 2015, 24: 800–809. 25861739 10.1007/s00586-015-3796-2

[47] Knight RQ , Schwaegler P , Hanscom D , Roh J . Direct lateral lumbar interbody fusion for degenerative conditions: early complication profile. J Spinal Disord Tech, 2009, 22: 34–37. 19190432 10.1097/BSD.0b013e3181679b8a

[48] Moller DJ , Slimack NP , Acosta FL Jr , Koski TR , Fessler RG , Liu JC . Minimally invasive lateral lumbar interbody fusion and transpsoas approach‐related morbidity. Neurosurg Focus, 2011, 31: E4. 10.3171/2011.7.FOCUS11137 21961867

[49] Silvestre C , Mac‐Thiong JM , Hilmi R , Roussouly P . Complications and morbidities of mini‐open anterior retroperitoneal lumbar interbody fusion: oblique lumbar interbody fusion in 179 patients. Asian Spine J, 2012, 6: 89–97. 22708012 10.4184/asj.2012.6.2.89 PMC3372554

[50] Lee HJ , Ryu KS , Hur JW , Seong JH , Cho HJ , Kim JS . Safety of lateral interbody fusion surgery without intraoperative monitoring. Turk Neurosurg, 2018, 28: 428–433. 28593626 10.5137/1019-5149.JTN.20103-17.1

[51] Miscusi M , Ramieri A , Forcato S , *et al* Comparison of pure lateral and oblique lateral inter‐body fusion for treatment of lumbar degenerative disk disease: a multicentric cohort study. Eur Spine J, 2018, 27: 222–228. 10.1007/s00586-018-5596-y 29671108

[52] Xu DS , Walker CT , Godzik J , Turner JD , Smith W , Uribe JS . Minimally invasive anterior, lateral, and oblique lumbar interbody fusion: a literature review. Ann Transl Med, 2018, 6: 104. 29707553 10.21037/atm.2018.03.24 PMC5900070

[53] Nakase H , Matsuda R , Tamaki R , Tei R , Park YS , Sakaki T . Two‐stage management for vertebral osteomyelitis and epidural abscess: technical note. Neurosurgery, 2006, 58: E1219 discussion E1219. 16723876 10.1227/01.NEU.0000215996.62828.76

[54] Pee YH , Park JD , Choi YG , Lee SH . Anterior debridement and fusion followed by posterior pedicle screw fixation in pyogenic spondylodiscitis: autologous iliac bone strut versus cage. J Neurosurg Spine, 2008, 8: 405–412. 18447685 10.3171/SPI/2008/8/5/405

[55] Shen X , Liu H , Wang G , *et al* The role of single‐stage posterior debridement, interbody fusion with titanium mesh cages and short‐segment instrumentation in thoracic and lumbar spinal tuberculosis. J Neurosurg Sci, 2017, 61: 473–480. 26149223 10.23736/S0390-5616.16.03333-6

